# Influence of proton irradiation on the magnetic properties of two-dimensional Ni(II) molecular magnet

**DOI:** 10.1038/s41598-023-41156-8

**Published:** 2023-08-28

**Authors:** Dominik Czernia, Piotr Konieczny, Ewa Juszyńska-Gałązka, Marcin Perzanowski, Janusz Lekki, Anabel Berenice González Guillén, Wiesław Łasocha

**Affiliations:** 1 Institute of Nuclear Physics PAN, ul. Radzikowskiego 152, 31-342 Cracow, Poland; 2 Research Center for Thermal and Entropic Science, Graduate School of Science, Osaka University, 1-1 Machikaneyamacho, Toyonaka, Osaka 560-0043 Japan; 3 Faculty of Chemistry, Jagiellonian University, ul. Gronostajowa 2, 30-387 Cracow, Poland

**Keywords:** Magnetic properties and materials, Phase transitions and critical phenomena

## Abstract

The influence of 1.9 MeV proton irradiation on structural and magnetic properties has been explored in the two-dimensional (2D) NiSO_4_(1,3-phenylenediamine)_2_ coordination ferrimagnet. The X-ray powder diffraction and IR spectroscopy revealed that the octahedrons with Ni ion in the center remain unchanged regardless of the fluence a sample received. In contrast, proton irradiation greatly influences the hydrogen bonds in the flexible parts in which the 1,3-phenylenediamine is involved. Dc magnetic measurements revealed that several magnetic properties were modified with proton irradiation. The isothermal magnetization measured at *T* = 2.0 K varied with the proton dose, achieving a 50% increase in magnetization in the highest measured field µ_0_*H*_dc_ = 7 T or a 25% decrease in remanence. The most significant change was observed for the coercive field, which was reduced by 90% compared to the non-irradiated sample. The observed results are accounted for the increased freedom of magnetic moments rotation and the modification of intralayer exchange couplings.

## Introduction

The recent intensive investigations in the field of multifunctional molecular materials are integrally related to their possible application in modern technology, such as high-density data storage and processing^1–4^, magnetic refrigeration^5–8^, or optically active sensors and switches^9,10^. The broad capability of molecular magnetism emerges from the variety of available structures^11,12^ and the possibility of changing their properties with external stimuli, including temperature, pressure^13^, and light irradiation^14^. Proton irradiation is another approach for tuning material properties by external factors. It is currently widely employed in the studies of carbon allotropes^15,16^, semiconductors^17^, films^18^, superconductors^19^, and alloys^20^ to engineer material features in a controllable manner^21–24^. Solids irradiated with energetic particles are exposed to extremely high-density local energy deposition that causes nonlinear and threshold effects^25^. As a result, new materials with novel properties can be obtained.

In particular, ion irradiation is used to modify magnetic properties. The induced defects may give rise to paramagnetic response in non-magnetic materials when the defect concentration exceeds a specific threshold value^26–28^ or alter the magnetic properties of paramagnets and systems with long-range magnetic order (LRMO), especially when strong magnetostructural correlations are present. The irradiation can increase Curie temperature^29–31^ and impact such properties as the coercive field, magnetization saturation, g-factor, and shape of the magnetic hysteresis loop^32–34^. Proton irradiation can generate changes at the atomic level over the selected area and depth by adjusting protons energy, which makes this approach perspective for the controlled engineering of magnetic materials^29^.

Currently, there are no systematic studies on the response of molecular magnetic materials to ion irradiation. To fill this gap, we examined the effects of 1.9 MeV proton irradiation on the magnetic properties of the NiSO_4_(1,3-phenylenediamine)_2_ coordination polymer, hereafter abbreviated as Ni(MPD)_2_SO_4_. This molecular magnet reveals a considerable size of the coercive field and long-range magnetic ordering below *T*_C_ = 24 K. X-ray powder diffraction, infrared spectroscopy, and magnetic measurements were performed to investigate the influence of proton fluence ϕ = 5 × 10^13^–2.2 × 10^15^ cm^−2^ on structural and magnetic properties. Although structural changes were observed only within the polymer part related to the C–H and N–H bonds, the proton irradiation has induced significant modifications of magnetic properties, especially the coercive field, degree of irreversibility, and maximum magnetization.

## Results

### X-ray powder diffraction (XRPD)

**Figure 1:**
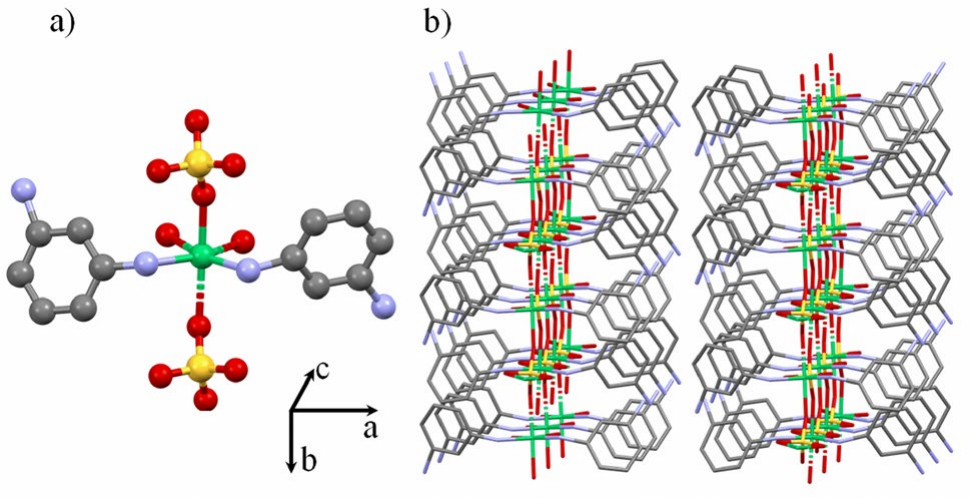
The crystal structure of Ni(MPD)_2_SO_4_: (**a**) representative molecular fragment (**b**) packing. Colors: Ni (green), S (yellow), O (red), C (black), N (light blue). Hydrogen atoms are omitted for clarity.

The Ni(MPD)_2_SO_4_ (Fig. 1) crystallizes in a monoclinic crystal system (*Cc* space group). The nickel (II) ions are bridged by tetrahedral ${\text{SO}}_{4}^{2 - }$ anions forming 2D layers parallel to the *bc* plane, and each layer is separated from one another by the 1,3-phenylenediamine (MPD) ligand. The nickel atom occupies the center of a distorted octahedron formed by two nitrogen atoms from the diamine molecules (MPD) and four oxygen atoms from three ${\text{SO}}_{4}^{2 - }$ anions. Each Ni^2+^ possesses six Ni^2+^ nearest neighbors and is separated from them by a distance of 4.914 Å, 4.931 Å, or 6.546 Å (two of each). The interlayer Ni–Ni distance is equal to 14.559 Å. More details regarding Ni(MPD)_2_SO_4_ structure were described in^35^. The XRPD measurements of all samples have not shown meaningful changes in the diffraction patterns pointing to the conservation of the Ni(MPD)_2_SO_4_ core structure after proton irradiation (see Supplementary Information Figure S1 online). However, greater half-widths of the diffraction peaks after proton irradiation may indicate the increase in crystal lattice microstrains or crystallites size reduction.

### Infrared spectroscopy (IR)

**Figure 2:**
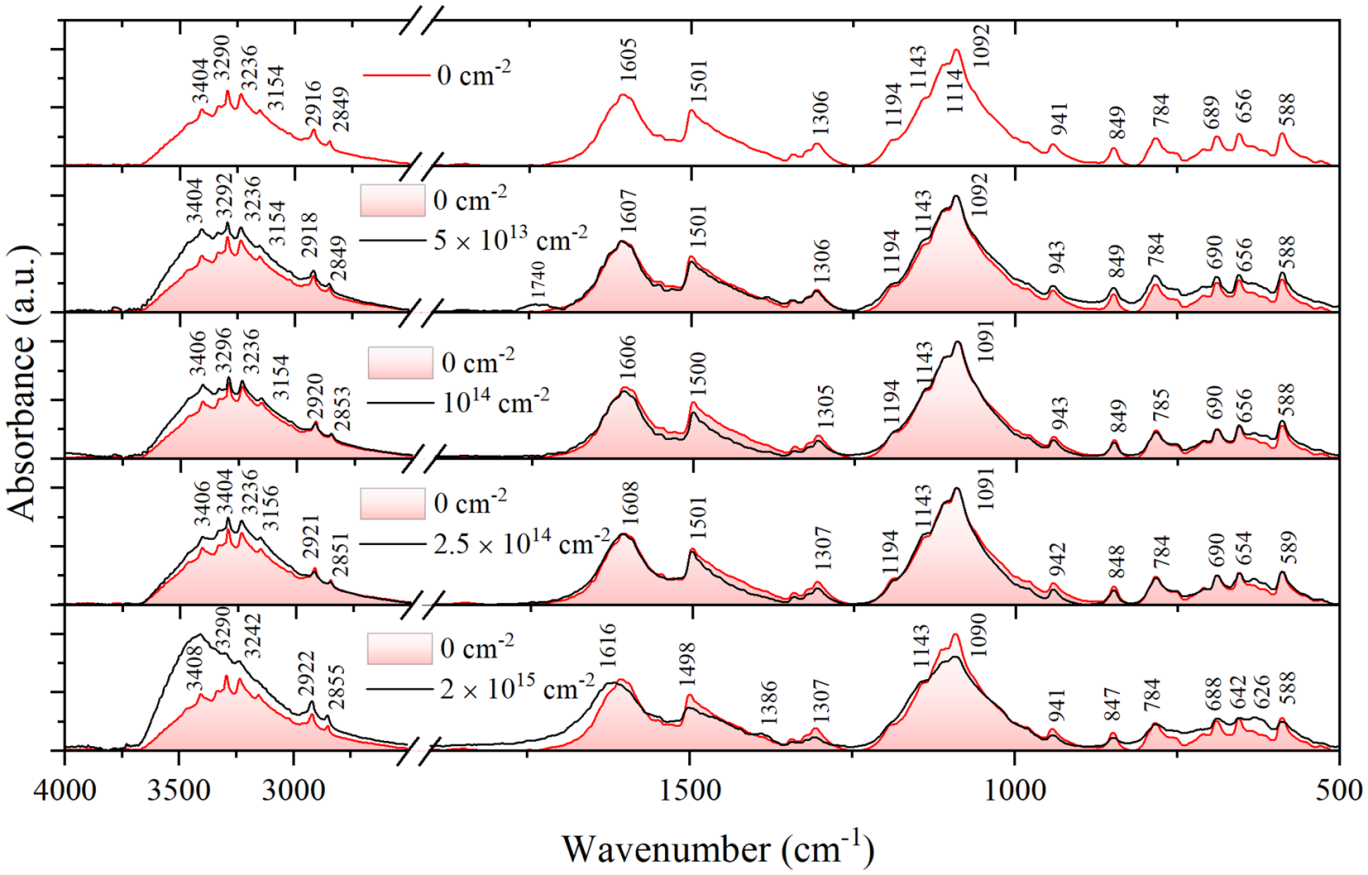
Fourier-transform infrared spectroscopy (FTIR) patterns of the Ni(MPD)_2_SO_4_ samples irradiated with various fluences.

In the mid-infrared range, the absorption spectra observed for Ni(MPD)_2_SO_4_, for samples irradiated with various fluences, have complex character (see Fig. 2). In the range of the deformation vibrations (ν < 2000 cm^−1^), a series of δ_C–H_ bands (in a ring), overlapping with those originating from SO_4_ ions, are visible. The IR spectra show NiSO_4_ sharp peaks of the characteristic S–O stretch [ν_S–O_(SO_4_) 900–1500 cm^−1^] and deformation [δ_S–O_(SO_4_) 400–700 cm^−1^] vibrations. On the other hand, a band corresponding to the stretching vibration from the Ni = O group is visible at about 600 cm^−1^. Broad bands, corresponding to S–O vibrations in the SO_4_ atomic group, were observed at about: ~ 525 cm^−1^ as δ_S–O_ deformation vibrations, and ~ 1100 cm^−1^ and ~ 1600 cm^−1^ as ν_S–O_ stretching vibrations^36^.

Vibration bands of the lighter atoms, i.e., C–H and N–H in MPD ligand, both deforming, i.e., in the range of 500 – 2000 cm^−1^, and the range of stretching vibrations, i.e., above 2000 cm^−1^, strongly overlap with the vibrations of the groups containing heavy atoms. They are visible in the form of low-intensity bands emerging from wide bands corresponding to the vibrations of the group, e.g., SO_4_. Bands at about (~ 700, ~ 1551) cm^−1^ are assigned to the vibrations of the CN group^37^.

The range of stretching vibrations deserves special attention. The broad band in this range (ν_CH_, ν_NH_) becomes asymmetric with the increase of the irradiation dose. The band is shifted towards a higher wavenumber (blue shift) which means that the strengths of the C–H and N–H are weakened^38^. The C–H and N–H bonds are involved well in forming hydrogen bonds, and the molecule's structure (shown in Fig. 1) perfectly enables it. A substantial change in the shape of the band with increasing irradiation dose proves the considerable influence of the hydrogen bonds on the ordering of the structure in its flexible parts in which MPD is involved.

Moreover, the visible asymmetry of the band indicates that there are different types of hydrogen bonds in which the nitrogen atom is involved. The nitrogen atom from the molecule's core (close to the nickel atom) is very stable (rigid) due to its involvement in covalent bonds. In contrast, the steric conditions for the "free" nitrogen atom favor the rapid formation/breaking of intermolecular bonds. Therefore, these weak interactions only affect the elastic part of the molecules, while the core of the molecule does not feel their presence. The assignments of major vibrational bands in the FTIR spectra of the Ni(MPD)_2_SO_4_ samples irradiated with various fluences are in Table 1.

**Table 1:** Assignment of major vibrational bands in the FTIR spectra of the Ni(MPD)_2_SO_4_ samples irradiated with various fluences^36–38^.

Wavenumber (cm^−1^)					Assignment
0 (cm^−2^)	5 × 10^13^ (cm^−2^)	10^14^ (cm^−2^)	2.5 × 10^14^ (cm^−2^)	2.2 × 10^15^ (cm^−2^)	
3404	3404	3406	3406	3408	ν_N–H_ (of free amines)
			3404		
3290	3292	3296		3290	
3236	3236	3236	3236	3242	
3154	3154	3154	3156		ν_C–H_ (in ring)
2916	2918		2921	2922	ν_C–H_ (in CH_2_)
2849	2849	2853	2851	2855	ν_C–H_ (in CH_2_)
	1740				
1605	1607	1606	1608	1616	δ_N-H_ (rocking vibrations)
1501	1501	1500	1501		
				1498	ν_CN_
				1386	ν_Ni–S_
1306	1306	1305	1307	1307	δ_C–H_
1194	1194	1194	1194		δ_C–C_
1143	1143	1143	1143	1143	δ_C–C_
1114	1114	1114	1114	1114	ν_SO4ˉ_
1092	1092	1091	1091	1090	ν_SO4ˉ_
941	943	943	942	941	δ_S–O_ + δ_C–H_
849	849	849	848	847	δ_S–O_ + δ_C–H_
784		785	784	784	δ_S–O_ + δ_C–H_
689	690	690	690	688	δ_S–O_ + δ_C–H_
656	656	656	654	642	δ_S–O_ + δ_C–H_
				626	δ_S–O_ + δ_C–H_
588	588	588	589	588	δ_S–O_ + ν_C–N_ + δ_C–H_

### Magnetic properties

**Figure 3:**
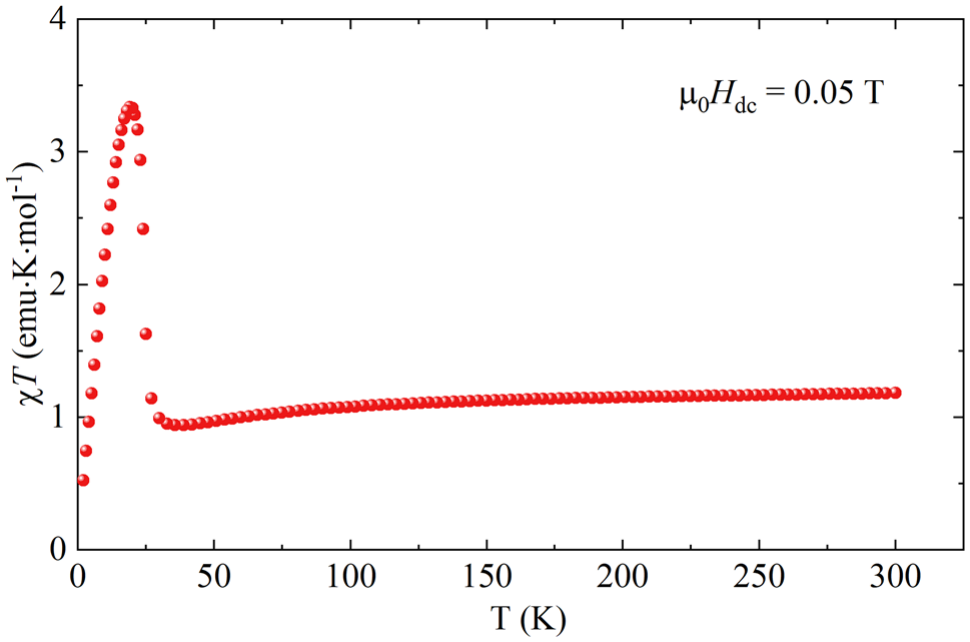
Temperature dependence of χ*T* product for Ni(MPD)_2_SO_4_ reference sample in the static magnetic field of µ_0_*H*_dc_ = 0.05 T.

The general magnetic properties of Ni(MPD)_2_SO_4_ were investigated in the recent work by González Guillén et al.^35^. Three magnetic couplings were found in the system: two competing intralayer superexchange pathways, antiferromagnetic and ferromagnetic, that lead to non-collinear ordering within a layer and a dipole–dipole coupling between layers. The low anisotropy of Ni(II) ions does not promote 2D long-range magnetic ordering (LRMO). Therefore, the LRMO occurs below the Curie temperature *T*_C_ = 24 K when all three interactions (where the dipolar coupling is the weakest one) are strong enough to couple magnetic moments in 3D. The product of molar magnetic susceptibility and temperature χ*T* of Ni(MPD)_2_SO_4_ measured in the temperature range *T* = 2.0 – 300 K in a constant magnetic field of µ_0_*H*_dc_ = 0.05 T is shown in Fig. 3. At *T* = 300 K, χ*T* equals 1.18 emu·K·mol^−1^, which can be obtained for a single isolated Ni^II^ ion with spin *S*_Ni_ = 1, assuming a spectroscopic factor *g* = 2.17. Such enhancement of *g* is expected for the Ni^II^ ion placed in the octahedral ligand field^39^.

**Figure 4:**
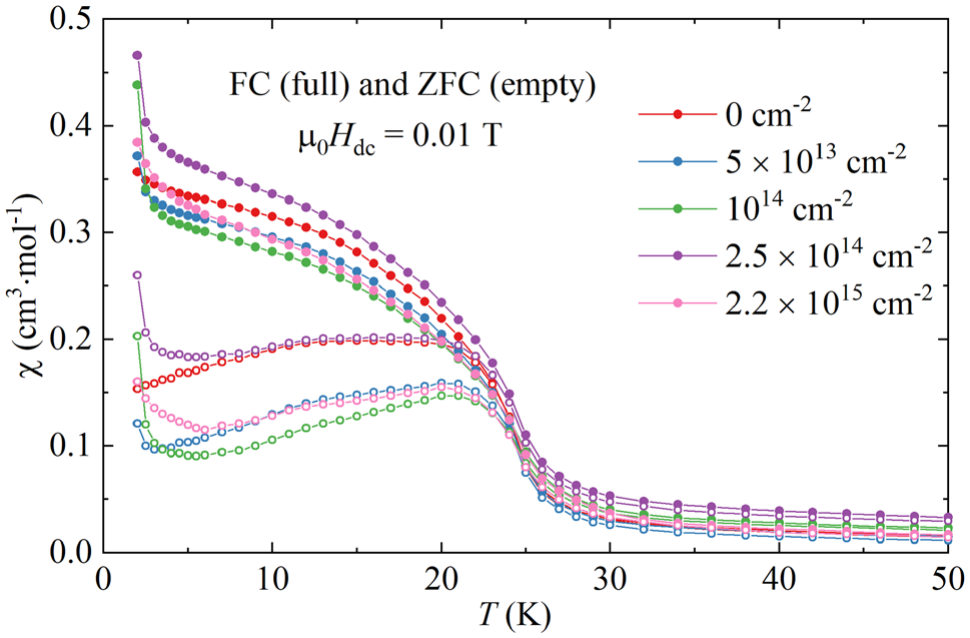
Field cooling (FC–full points) and zero-field cooling (ZFC – empty points) magnetic susceptibilities of Ni(MPD)_2_SO_4_ samples irradiated with various fluences ϕ measured in static magnetic field µ_0_*H*_dc_ = 0.01 T at *T* = 2.0–50 K.

The field cooling (FC) and zero-field cooling (ZFC) magnetic susceptibilities of Ni(MPD)_2_SO_4_ as a function of temperature are shown in Fig. 4 for proton fluences ranging from ϕ = 0 cm^−2^ to ϕ = 2.2 × 10^15^ cm^−2^. The temperature of phase transition to LRMO obtained from the temperature derivatives of FC and ZFC curves was *T*_C_ = 24 K regardless of the sample radiation dosage. Compared to the reference sample, an additional increase in susceptibility was observed at the lowest temperatures for the samples irradiated with protons. However, the overall shapes of FC/ZFC were preserved above about *T* = 5.0 K. The obtained susceptibility magnitude varied between each sample; for the FC mode, from 90 to 130% of the reference sample’s corresponding value, whereas for ZFC, it varied between 55 and 170%. The largest FC/ZFC signal was obtained for ϕ = 2.5 × 10^14^ cm^−2^ and the lowest for ϕ = 10^14^ cm^−2^. The former sample was also the only one for which the FC/ZFC values above *T* = 5.0 K were greater than the values for the reference sample.

The pair of ZFC and FC curves coincide with each other only above a certain irreversibility temperature *T*_irr_^40^, which can be found by plotting the difference between FC, χ_FC_(*T*), and ZFC, χ_ZFC_(*T*), susceptibilities, and determining the temperature above which the difference becomes less than 5%. The degree of irreversibility Δχ_irr_ was estimated for each studied fluence using the following integral^41^:

1 $$\Delta \chi_{\texttt {irr}} = \mathop \smallint \limits_{T_{1} }^{T_{2} } ( {\chi_{\texttt {FC}} \left( T \right) - \chi_{\texttt {ZFC}} (T)}) \texttt{d} \it T,$$

where *T*_1_ = 2.0 K and *T*_2_ = *T*_irr_.

**Figure 5:**
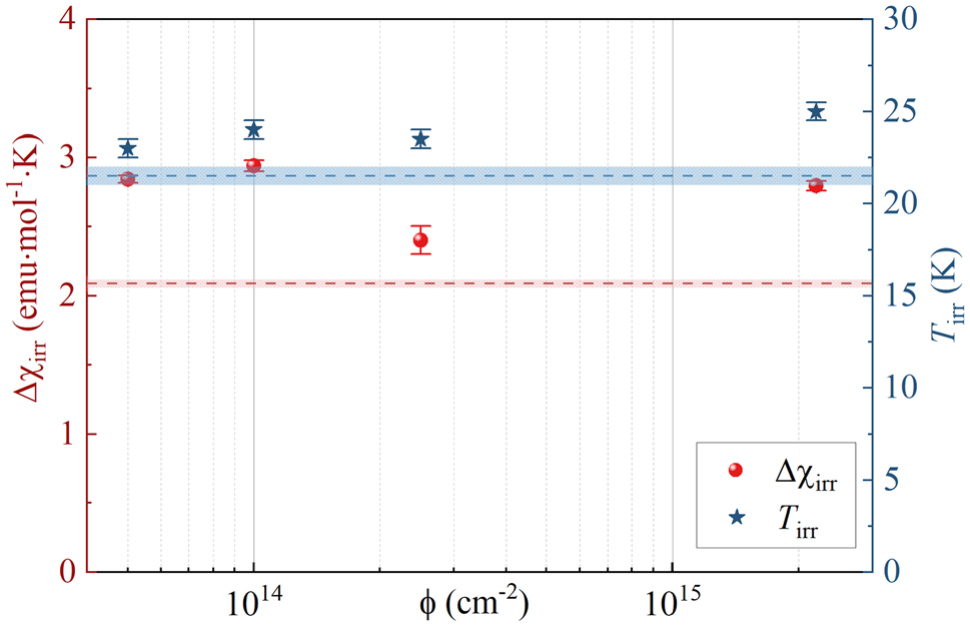
The degree of irreversibility Δχ_irr_ (1) (left axis) and irreversibility temperature *T*_irr_ (right axis) for Ni(MPD)_2_SO_4_ samples irradiated with various fluences. The dashed lines represent the values obtained for the reference sample ϕ = 0 cm^−2^ and dotted areas their errors.

The *T*_irr_ and ∆χ_irr_ are summarized in Fig. 5 for each irradiation dose. The *T*_irr_ weakly depends on the irradiation dose revealing values between 23 and 25 K, corresponding to 105–115% of the reference sample’s *T*_irr_. However, proton irradiation has a stronger influence on the degree of irreversibility. The obtained values of Δχ_irr_, between 2.4 emu·mol^−1^·K and 2.95 emu·mol^−1^·K, are from 115 to 140% of the reference sample’s Δχ_irr_. The highest *T*_irr_ = 25 K was found for ϕ = 2.2 × 10^15^ cm^−2^, while the maximal value of Δχ_irr_ = 2.95 emu·mol^−1^·K was for ϕ = 10^14^ cm^−2^.

**Figure 6:**
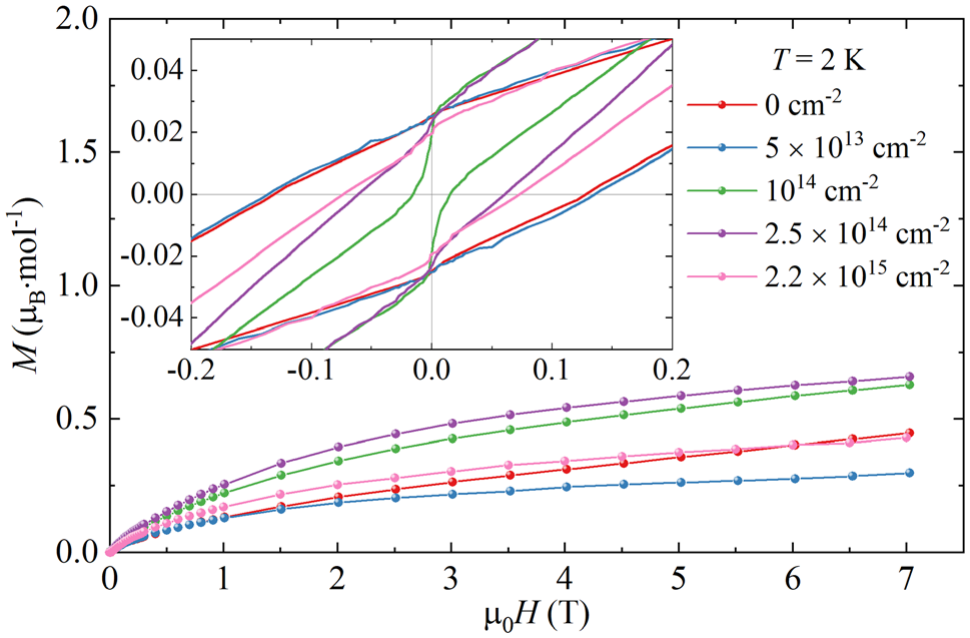
The isothermal magnetization of Ni(MPD)_2_SO_4_ samples irradiated with various fluences ϕ measured at *T* = 2.0 K and in applied magnetic fields from zero to µ_0_*H* = 7 T. Inset: the picture enlarged on µ_0_*H* = [− 0.2 T, 0.2 T] range to show the coercivity and remanence of the samples.

The Fig. 6 shows the isothermal magnetization of Ni(MPD)_2_SO_4_ obtained at *T* = 2.0 K for all studied fluences ϕ. In µ_0_*H* = 7 T, the lowest value of magnetization *M*_7T_ = 0.3 μ_B_·mol^−1^ was observed for ϕ = 5 × 10^13^ cm^−2^ sample and the highest *M*_7T_ = 0.65 μ_B_·mol^−1^ for ϕ = 5 × 10^14^ cm^−2^ sample, compared to *M*_7T_ = 0.45 μ_B_·mol^−1^ for the reference sample (where *M*_7T_ is the magnetization at µ_0_*H* = 7 T).

**Figure 7:**
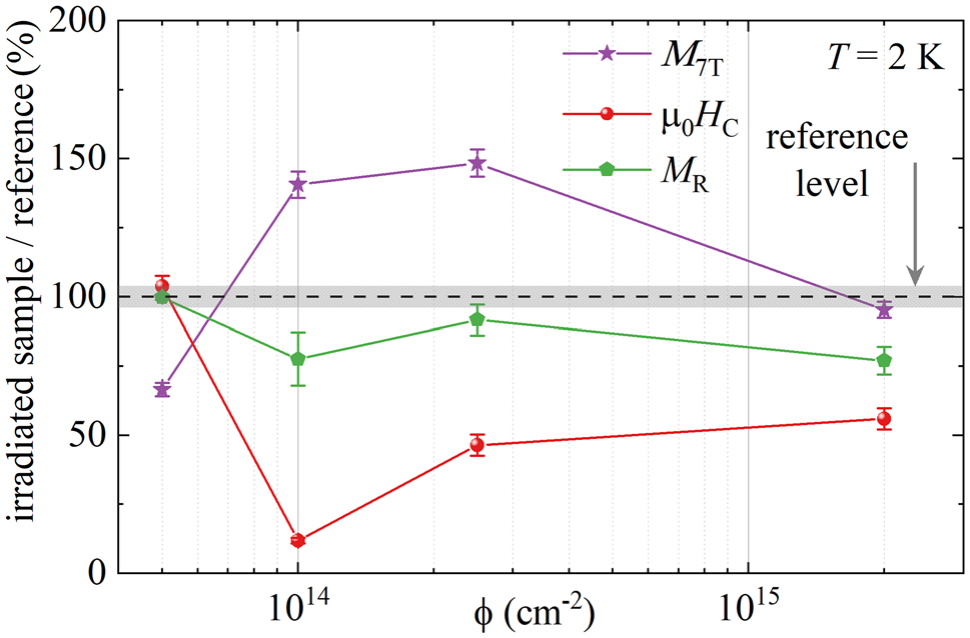
The relative values of magnetic hysteresis loop parameters for Ni(MPD)_2_SO_4_ samples irradiated with various fluences ϕ normalized to the reference sample’s corresponding values. Violet stars: magnetization at µ_0_*H* = 7 T (*M*_7T_); red circles: coercive field (µ_0_*H*_c_); green pentagons: remnant magnetization (*M*_R_). The dashed line is placed at 100% and represents the values obtained for the reference sample ϕ = 0 cm^−2^ with errors indicated by gray dotted area. The lines between points are only for an eye guide.

The low field part of magnetic hysteresis loops is depicted in the inset of Fig. 6, enlarged on the coercivity µ_0_*H*_c_ and remanence *M*_R_. The *M*(*H*) loops were opened below µ_0_*H* = 1 T for all the samples. The hysteresis loop for the sample with the lowest fluence of ϕ = 5 × 10^13^ cm^−2^ coincided with the reference’s loop in the field range µ_0_*H* = [− 1 T, 1 T]. For the remaining samples, the values of µ_0_*H*_c_ and *M*_R_ were reduced, and a step in the *M*(*H*) loop near µ_0_*H* = 0 T appeared, which is especially visible for ϕ = 10^14^ cm^−2^. Figure 7. shows the hysteresis loop parameters for the irradiated samples, presented as a relative value to the reference sample’s coercivity (µ_0_*H*_c_ = 0.13 T), remanence (*M*_R_ = 0.45 μ_B_·mol^−1^), and magnetization at µ_0_*H* = 7 T (*M*_7T_ = 0.3 μ_B_·mol^−1^). *M*_7T_ is reduced for ϕ = 5 × 10^13^ cm^−2^ to 65% of the reference level, but it raises with an increasing ϕ to 150% for ϕ = 2.5 × 10^14^ cm^−2^. The further increase in fluence reduces the value of *M*_7T_, which becomes 95% of the reference level for the sample with the highest studied fluence of ϕ = 2.2 × 10^15^ cm^−2^. The samples irradiated with the fluences ϕ ≥ 10^14^ cm^−2^ showed the reduction of µ_0_*H*_c_ to 10–55% and *M*_R_ to 75–90% of the reference level.

## Discussion

The XRPD and IR spectroscopy measurements indicated that the core structure of Ni(MPD)_2_SO_4_ remains unchanged. Consequently, the interlayer distance and the dipolar interaction are the same regardless of the fluence a sample received. Furthermore, the Curie temperature *T*_C_ remains the same because its value is determined by the weakest magnetic coupling, the dipole–dipole one. On the other hand, irradiation significantly modifies the C–H and N–H bonds, which are involved in forming hydrogen bonds related to the MPD ligand (the flexible part of the structure). In particular, the interaction of lattice with protons may lead to the C–H and N–H bonds breakage, creating hydrogen vacancies that would not be visible in XRPD, as reported in the methylammonium lead iodide perovskites after 10 MeV proton irradiation^42^ or polymer composite of polymethyl methacrylate doped with ferric oxalate after 3 MeV proton irradiation^43^.

The bond modification leads to changes in the crystal field, incorporation of local defects, altered intralayer exchange couplings (due to local structure modification), and increased freedom of the magnetic moments. In consequence, magnetic properties in the LRMO phase (below *T*_C_) have been modified. Table 2. summarizes the influence of proton irradiation on the magnetic properties of Ni(MPD)_2_SO_4_.

**Table 2:** The influence of proton irradiation on *T*_irr_, Δχ_irr_, *M*_7T_, *M*_R_, and µ_0_*H*_c_ in Ni(MPD)_2_SO_4_. Absolute values and their ratio to the corresponding values for the reference sample (in percentages) are given.

Φ Fluence (cm^−2^)	*T*_irr_		Δχ_irr_		*M*_7T_		*M*_R_		µ_0_*H*_c_	
	(K)	%	(emu·mol^−1^·K)	%	(μ_B_·mol^−1^)	%	(μ_B_·mol^−1^)	%	(Oe)	%
0 (ref.)	21.5	100	2.09	100	0.45	100	0.025	100	1310	100
5 × 10^13^	23	107	2.84	136	0.3	67	0.025	99	1360	104
10^14^	24	112	2.94	141	0.63	141	0.02	78	155	12
2.5 × 10^14^	23.5	109	2.4	115	0.69	148	0.023	92	265	46
2.2 × 10^15^	25	116	2.8	134	0.43	95	0.019	77	735	56

The isothermal magnetization measurements (Fig. 6) confirm a strong influence of irradiation on the magnetization processes in Ni(MPD)_2_SO_4_. The main reason for this is the increasing number of defects with increasing fluence. Note that irradiating the samples with the selected fluences is not enough to obtain a homogenous distribution of produced defects, and the observed magnetization data may be a mixture of several defect-induced magnetic phases of Ni(MPD)_2_SO_4_ with well-localized clusters. Introducing defects lowers the magnetostatic energy to a minimum for the dose of ϕ = 10^14^ cm^−2^ (the lowest value of the coercive field). However, a further increase in the number of defects leads to stronger pinning effects, which increase the coercive field for doses ϕ > 10^14^ cm^−2^.

The mechanism of *M*_7T_ modification is different and related to changes in magnetic couplings in the compound. For the lowest dose of ϕ = 5 × 10^13^ cm^−2^, antiferromagnetic interactions are more dominant over the ferromagnetic ones, which lowers the magnetization *M*_7T_ down to 67% of the *M*_7T_ for the reference sample. For intermediate doses of ϕ = 10^14^ cm^−2^ and ϕ = 2.5 × 10^14^ cm^−2^, the corresponding values are 141% and 148%, respectively, indicating the enhancement of ferromagnetic interactions, and for the maximum dose of ϕ = 2.2 × 10^15^ cm^−2^, the value of *M*_7T_ is similar to that for the reference. Note that the calculated magnetization for the parallel alignment of Ni^II^ magnetic moments (*S*_Ni_ = 1) with a spin-only Landé factor of *g* = 2.17^35,39^ yields the magnetization saturation of *M*_S_ = 2.17 µ_B_·mol^−1^. Thus, the antiferromagnetic interactions are dominant in all studied samples.

Similar non-monotonic effects of proton irradiation are commonly found in the literature^31,33,34,44–46^. In the Ni_48·4_Mn_28.8_Ga_22.8_ film irradiated with 120 keV protons^45^, the *M*_S_ for ϕ = 10^15^ cm^−2^ raised to 155% of the non-irradiated sample’s value, but for ϕ = 2 × 10^16^ cm^−2^, the observed *M*_S_ was only 102%. Another case is the initially diamagnetic SiC (0001) single crystal^44^ that displays ferromagnetism after 3 MeV proton irradiation treatment. For ϕ = 8 × 10^15^ cm^−2^, the *M*_S_ measured in-plane reached 0.17 emu·g^−1^ but for ϕ = 1.3 × 10^16^ cm^−2^ it was reduced to 0.09 emu·g^−1^.

An additional increase of the magnetization in the FC/ZFC curves observed at *T* = 2–6 K for all irradiated samples may arise from a small sample’s fraction, which due to the deformation and partial destruction of a lattice, leads to the creation of localized magnetic moments due to open bonds^47^ or paramagnetic amorphous zones in the lattice^44^. This paramagnetic contribution may also cause the appearance of the observed step in the hysteresis loop near-zero field. The other possibility is the chemical composition modification of Ni(MPD)_2_SO_4_ samples by incorporating the highly energetic hydrogen atoms into the crystal lattice^44,45^. However, no additional phases were detected in the XRPD and IR measurements. Therefore, the amount of paramagnetic phase is not significant.

## Conclusions

In summary, we have investigated the impact of 1.9 MeV proton irradiation on the layered Ni(MPD)_2_SO_4_ molecular magnet in the function of received fluence ϕ. It was found that proton irradiation influences only the flexible part of the structure (related to the C–H and N–H bonds in the MPD ligand), while the core of the compound remains unchanged. These lead to significant changes in magnetic properties as a function of fluence: coercive field µ_0_*H*_c_ (reduction up to 90%), maximal magnetization *M*_7T_ (between 65 and 150% of the reference sample), remanence *M*_R_ (reduction up to 25%) and degree of irreversibility Δχ_irr_ (increase up to 40%). Simultaneously, the Curie temperature was constant *T*_C_ = 24 K regardless of ϕ because the core structure, interlayer distance, and, consequently, dipolar coupling of the Ni(MPD)_2_SO_4_ system remains the same. The observed non-monotonic magnetic properties changes are caused by the altered intralayer exchange couplings due to local structure modification, incorporation of local defects, and increased freedom of the magnetic moments.

## Materials and methods

### Sample preparation

The studied Ni(C_6_H_8_N_2_)_2_SO_4_ compound (hereafter abbreviated as Ni(MPD)_2_SO_4_) was synthesized using NiSO_4_·7H_2_O and 1,3-phenylenediamine (MPD, C_6_H_8_N_2_) ligand with a solvent-free synthesis approach according to the literature procedure^35^.

The Van de Graaff accelerator was used to generate a proton beam with set conditions: energy of 1.9 MeV, particle current of 10–12 nA, and beam size of about 0.5 mm × 0.5 mm. Powder samples mounted on copper plates were used (see Supplementary Figure S2 online) with a thickness lower than the 1.9 MeV proton penetration depth (see Supplementary Figure S3 online). The obtained fluences were equal to: ϕ = 5 × 10^13^ cm^−2^, 10^14^ cm^−2^, 2.5 × 10^14^ cm^−2^, 2.2 × 10^15^ cm^−2^.

### Characterization methods

X-ray powder diffraction (XRPD) patterns were determined by the PANalytical X’Pert Pro instrument with a copper X-ray tube source (Cu K _α1_, 1.541 Å), operated at 40 kV and 30 mA in the θ–2θ geometry. Data were recorded at room temperature in the range of 5–40° 2θ. The results were adjusted by background correction and the anode’s characteristic X-ray K_α2_ removal.

The infrared spectroscopy measurements were done for samples in powder form, pelleted with potassium bromide (transparent in the mid-infrared range). Fourier-transform infrared spectroscopy (FTIR) measurements were carried out in the mid-infrared range (4000–500 cm^−1^), with a resolution of 2 cm^−1^, on the EXCALIBUR 3000 spectrometer; 128 scans were done for the spectrum at room temperature.

The Quantum Design SQUID MPMS-XL magnetometer was used to measure the magnetic properties of all powder samples. The isothermal magnetization *M*(µ_0_*H*) was collected in the applied field of µ_0_*H* = [− 7 T, 7 T]. Static magnetic susceptibility measurements were carried out in the field cooling (FC) and zero-field cooling (ZFC) modes in the T = 2.0–300 K temperature range with the applied field of µ_0_*H*_dc_ = 0.01 T. The obtained data points were processed taking the whole sample mass, i.e., assuming a homogeneous distribution of potential changes in the studies compounds after proton irradiation. The temperature-independent diamagnetic contribution of χ_0_ =  − 1.84 × 10^−4^ emu·mol^−1^ estimated from Pascal’s constants^48^ was subtracted from the measured signals.

### Supplementary Information

Supplementary Information.

## Data Availability

The datasets used and/or analyzed during the current study are available from the corresponding author on reasonable request.
