# Supplementary material for: Influence of proton irradiation on the magnetic properties of two-dimensional Ni(II) molecular magnet
Source: Sci Rep. 2023 Aug 28;13:14032. doi: 10.1038/s41598-023-41156-8 (PMC10462683; doi:10.1038/s41598-023-41156-8)
Supplement: Supplementary file 1 — Supplementary Information. [file 41598_2023_41156_MOESM1_ESM.docx]

**Influence of proton irradiation on the magnetic properties of two-dimensional Ni(II) molecular magnet**

**Supplementary Information**

Dominik Czernia^a^*, Piotr Konieczny^a^*, Ewa Juszyńska-Gałązka^a,b^, Marcin Perzanowski^a^, Janusz Lekki^a^, Anabel Berenice González Guillén^c^, Wiesław Łasocha^c^

^a^Institute of Nuclear Physics PAN, ul. Radzikowskiego 152, 31-342 Cracow, Poland

^b^Research Center for Thermal and Entropic Science, Graduate School of Science, Osaka University, 1-1 Machikaneyamacho, Toyonaka, Osaka 560-0043, Japan

^c^Faculty of Chemistry, Jagiellonian University, ul. Gronostajowa 2, 30-387 Cracow, Poland

*Corresponding authors: [dominik.czernia@ifj.edu.pl](mailto:dominik.czernia@ifj.edu.pl), [piotr.konieczny@ifj.edu.pl](mailto:piotr.konieczny@ifj.edu.pl)

1. **X-ray powder diffraction (XRPD)**

In Figure 1., the XRPD patterns for unirradiated (φ = 0 cm^-2^) and the representative irradiated (φ = 2.5 × 10^14^ cm^-2^) samples were compared, showing the presence of the same peaks and the coincidence of their positions. Therefore, no significant changes have been observed in the structure of the Ni(C_6_H_8_N_2_)_2_SO_4_ compound (**Ni(MPD)_2_SO_4_**) after 1.9 MeV proton irradiation. Similar conclusions can be drawn for the rest of the irradiated samples.


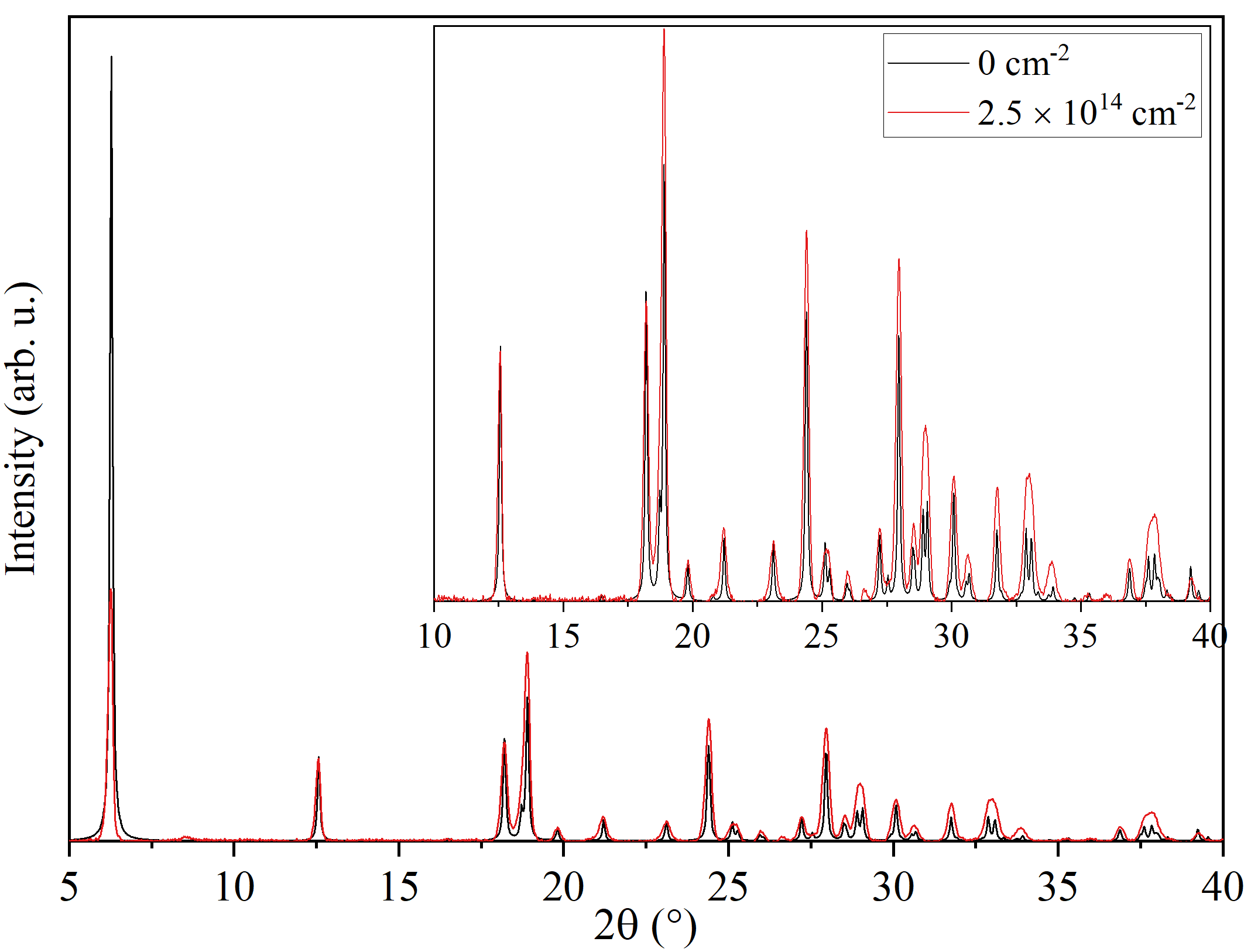


Supplementary Figure S1. Normalized X-ray powder diffraction pattern for the **Ni(MPD)_2_SO_4_** reference sample (black line) and the sample irradiated with the fluence of φ = 2.5 × 10^14^ cm^-2^ (red line) for 2θ ranging from 5° to 40°. Inset: the picture enlarged at the 10° to 40° range.

1. **Sample preparation**

Powder samples of **Ni(MPD)_2_SO_4_** were evenly distributed on copper plates covering the area of 5 × 5 mm^2^ to obtain a uniform layer of crystallites. The layer was tightly covered with Goodfellow’s polyethylene terephthalate foil with a thickness of 0.9 µm and glued with Varnish GE adhesive (Fig. 2). The copper plate’s purpose was to gather an excess of electrical current from incoming protons. The foil was used to secure the powder from vacuum and movement due to the copper plate’s vertical rotation. The powder samples were removed from the copper plates after the irradiation, weighted, and transported to the new sample holder (a plastic straw) for the magnetometry measurements.





Supplementary Figure S2. The samples of **Ni(MPD)_2_SO_4_** (black powder) prepared for irradiation on copper plates covered with thin foil.

1. **Proton irradiation**

The 1.9 MeV proton penetration range of about 46.5 µm in the NiSO_4_C_12_H_16_N_4_ target (Fig. 3a) was computed employing simulations from the SRIM code [1]. It exceeds the estimated thickness of 30 µm of the sample layer and foil in the experiment to avoid proton implantation. It also ensures relatively uniform irradiation through the whole sample’s volume (Fig. 3b). Six copper plates with powder samples of **Ni(MPD)_2_SO_4_** were mounted on the movable holder and homogeneously irradiated for varying times.


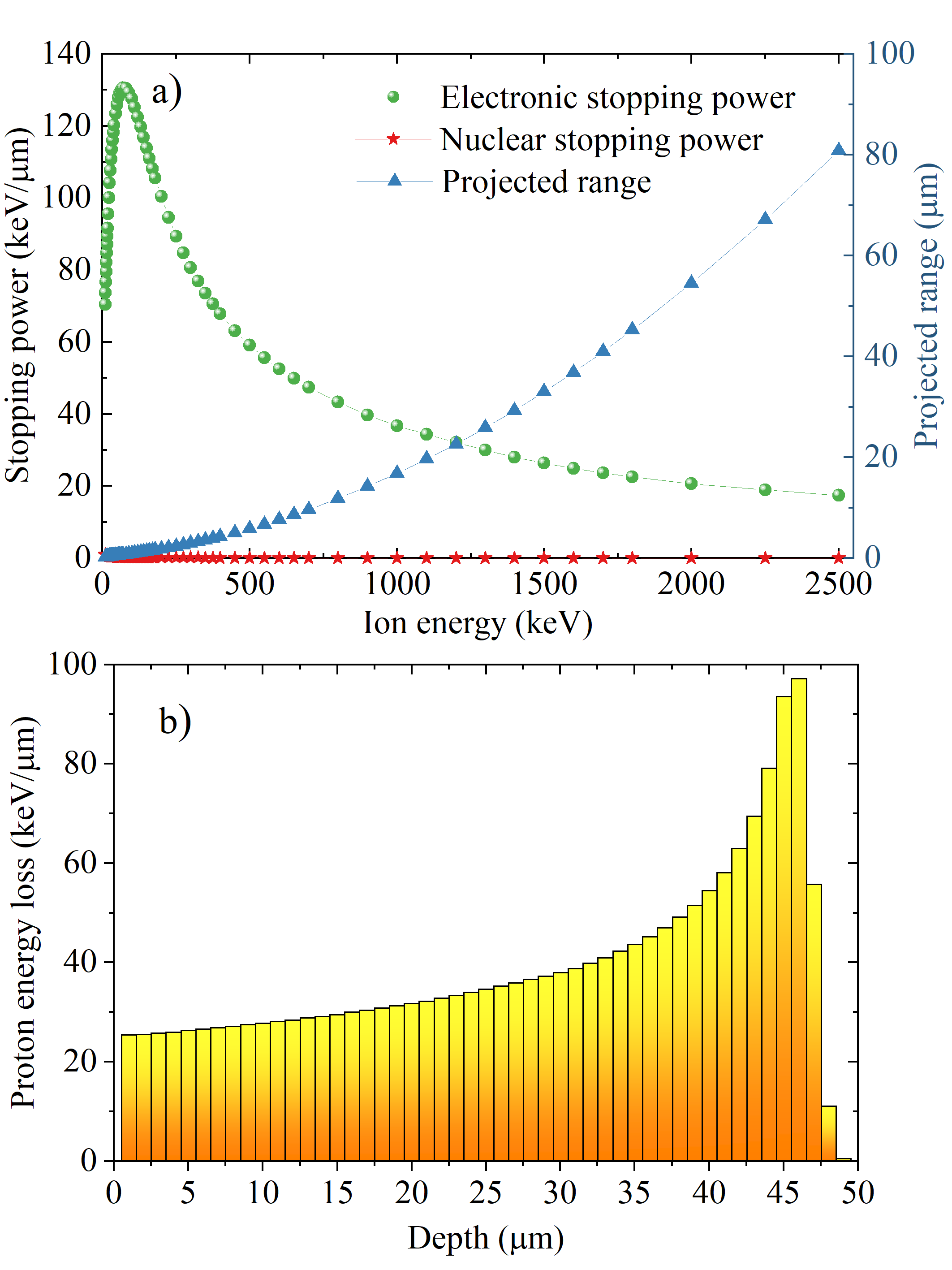


Supplementary Figure S3. (a) On the left axis, electronic and nuclear stopping powers as a function of proton energy up to 2.5 MeV moving in the NiC_12_H_16_N_4_SO_4_ target. The right axis shows the projected range of implemented protons. (b) Energy distribution for NiC_12_H_16_N_4_SO_4_ target for 1.9 MeV protons over the implantation depth.

1. **References**

1. Ziegler, J. F., Ziegler, M. D. & Biersack, J. P. SRIM - The stopping and range of ions in matter (2010). *Nucl. Instruments Methods Phys. Res. Sect. B Beam Interact. with Mater. Atoms* **268**, 1818–1823 (2010).
